# Crosstalk between endocannabinoid and immune systems: a potential dysregulation in depression?

**DOI:** 10.1007/s00213-015-4105-9

**Published:** 2015-10-20

**Authors:** Emily Boorman, Zuzanna Zajkowska, Rumsha Ahmed, Carmine M. Pariante, Patricia A. Zunszain

**Affiliations:** Stress, Psychiatry and Immunology Laboratory, Institute of Psychiatry, Psychology & Neuroscience, King’s College London, London, UK

**Keywords:** Inflammation, Cytokine, CNS, Lymphocyte, Eicosanoids, Prostaglandin, Microglia, Microglial polarisation, Anandamide, 2-AG

## Abstract

**Background:**

The endocannabinoid (eCB) system, an endogenous lipid signaling system, appears to be dysregulated in depression. The role of endocannabinoids (eCBs) as potent immunomodulators, together with the accumulating support for a chronic low-grade inflammatory profile in depression, suggests a compelling hypothesis for a fundamental impairment in their intercommunication, in depression.

**Objective:**

We aim to review previous literature on individual associations between the immune and eCB systems and depression. It will focus on peripheral and central mechanisms of crosstalk between the eCB and immune systems. A potential dysregulation in this crosstalk will be discussed in the context of depression.

**Results:**

Investigations largely report a hypoactivity of the eCB system and increased inflammatory markers in individuals with depression. Findings depict a multifaceted communication whereby immunocompetent and eCB-related cells can both influence the suppression and enhancement of the other’s activity in both the periphery and central nervous system. A dysregulation of the eCB system, as seen in depression, appears to be associated with central and peripheral concentrations of inflammatory agents implicated in the pathophysiology of this illness.

**Conclusion:**

The eCB and immune systems have been individually associated with and implicated in pathogenic mechanisms of depression. Both systems tightly regulate the other’s activity. As such, a dysregulation in this crosstalk has potential to influence the onset and maintenance of this neuropsychiatric illness. However, few studies have investigated both systems and depression conjointly. This review highlights the demand to consider joint eCB-immune interactions in the pathoetiology of depression.

## Introduction

An increasing number of pathways external to the brain are gaining support in explaining the pathogenesis of major depressive disorder (MDD). In particular, findings suggest an involvement of immunedysregulation (Maes et al. 1995a; Raison et al. 2006; Dantzer et al. 2008; Dowlati et al. 2010; Zunszain et al. 2013) and alterations of the endocannabinoid (eCB) signalling system (Hillard and Liu 2014) in the onset and maintenance of this mood disorder.

Research has supported the involvement of immunedysregulation and more specifically, a low-grade inflammation, in depression (Berk et al. 2013). Findings reveal heightened levels of inflammatory markers present in the peripheral blood and cerebral spinal fluid (CSF) of depressed individuals, compared to healthy controls. The influences of this chronic inflammatory state have been implicated in virtually every neuropathophysiologic abnormality associated with major depression (Miller et al. 2009).

**Fig. 1:**
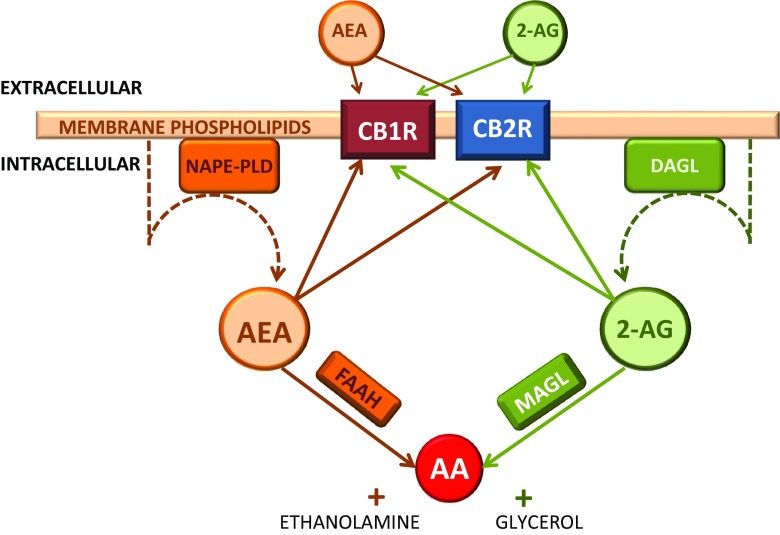
AEA and 2-AG are synthesised from enzymes, NAPE-PLD and DAGL, respectively, from glycerophospholipids in the phospholipid bilayer of the cellular membrane. Both eCBs bind to intracellular and extracellular CB1R and CB2R. Intracellular degradation of AEA and 2-AG occurs when bound to FAAH and MAGL, respectively. Products of this are AA and either glycerol, from 2-AG, or ethanolamine, from AEA

The involvement of the eCB system in MDD has been suggested more recently. The eCB system is a homeostatic mechanism identified following the discovery of the main psychoactive constituent in marijuana, ∆^9^-tetrahydrocannabinol (THC) (Gaoni and Mechoulam 1964). This system refers to three main components: (1) the endogenous lipid transmitters: endocannabinoids (eCBs) anandamide (AEA) and 2-arachidonoylglycerol (2-AG) (Devane et al. 1992; Mechoulam et al. 1995); (2) the target of these eCBs: G-protein-coupled receptors, cannabinoid receptor type 1 (CB1R) and 2 (CB2R) (Matsuda et al. 1990; Munro et al. 1993); and (3) enzymes involved in the synthesis, namely *N*-acylphosphatidylethanolamine-hydolyzing phospholipase D (NAPE-PLD) and diacylglycerol lipase (DAGL) (Bisogno et al. 2003; Okamoto et al. 2004) and degradation enzymes including fatty acid amide hydrolase (FAAH) and monoacylglycerol lipase (MAGL) (Cravatt et al. 1996; Karlsson et al. 1997). Generated from membrane-bound glycerophospholipids (Wang and Ueda 2009), eCBs refer to the wide spectrum of lipid-derived fatty acid structural analogues (Alexander and Kendall 2007) of which AEA and 2-AG are the best described (Fig. 1). These naturally occurring cannabinoids present similar psychoactive properties to THC. They act on both receptors CB1R and CB2R. There is an increasing interest into eCB-like molecules that converge on common catabolic and metabolic pathways, notably non-cannabinoid AEA structural analogues, palmitoylethanolamine and oleoylethanolamine (Alexander and Kendall 2007). Involvement of alternative targets, non-classical receptors, such as peroxisome proliferator-activated receptor (PPAR)-α and transient receptor potential cation channel sub-family V member 1 (TRPV1), are also being investigated. However, investigations into associations between the aforementioned and depression are scarce. As such, influences of 2-AG and AEA will make up the evidence base for this discussion.

Interestingly, there is a clear divide in expression levels between tissues, with CB1R being expressed most commonly in the brain and CB2R principally located across an array of immune cells in the periphery (Malfitano et al. 2014). CB1R and CB2R have thus shown differential involvement in neuromodulation and immunoregulation, respectively (Castillo et al. 2012).

This review will focus on the possible associations between the immune and eCB systems and depression, both independently of each other and interdependently. It will highlight pathophysiological changes associated with chronic activation of the immune system that may be mechanistically relevant in facilitating the pathogenesis and potentiation of this neuropsychiatric disorder. As such, individual involvement of eCB activity and inflammation in depression will be outlined initially, followed by an examination of the evidence relevant to their intercommunication, in the context of depression.

## The inflammatory model of depression

Recent meta-analyses provide prominent support for an association between immune dysfunction and depression. Patients with MDD showed elevated levels of several inflammatory signalling proteins, such as IL-6, IL-1, tumour necrosis factor-alpha (TNF-α) and C-reactive protein (CRP), in both serum and plasma (Howren et al. 2009; Dowlati et al. 2010). Moreover, a dose-response relationship has been observed between severity of symptoms and levels of pro-inflammatory cytokines as measured in a population continuum from community-based individuals to those clinically depressed (Howren et al. 2009). Furthermore, exogenous administration of the pro-inflammatory cytokine interferon (IFN)-α, a recommended immunotherapeutic treatment for chronic hepatitis C viral infection, leads to the onset of depression in around 30 % of these patients (Asnis and De La Garza 2006). This is believed to be as a result of the increased inflammatory response (McNamara and Lotrich 2012). A close relationship has also been reported between dose of IFN-α and subsequent changes in mood (Baraldi et al. 2012).

Further support comes from reports depicting an increasingly evident comorbidity between depression and inflammatory conditions, particularly those of an autoimmune-nature (Iseme et al. 2014). A study measuring the prevalence of comorbidity between the inflammatory disease, psoriasis and depression, found that 40 % of individuals diagnosed score positively for depressed mood and 27 % were diagnosed with MDD (Zaher et al. 2010). Interestingly, in a subsequent investigation, etanercept, a biopharmaceutical with anti-inflammatory properties, significantly reduced the depressive symptomatology presented in the same patient group, independently of any improvements in the condition (Tyring et al. 2006). Other anti-inflammatory drugs, including celecoxib (a cyclooxygenase-2 inhibitor), minocycline and acetyl salicylic acid, have been similarly effective as adjunctive therapies in reducing depressive symptoms (Mendlewicz et al. 2006; Soczynska et al. 2012; Maciel et al. 2013; Köhler et al. 2014). Interestingly, current psychotropic treatments have also been seen to possess anti-inflammatory qualities, suggesting a possible contribution of immunoregulation in their efficacy (Maes et al. 1999; Cattaneo et al. 2013; Horowitz et al. 2014).

Microglia activation is one of the mechanisms by which peripheral immune challenges can alter brain functioning (Dantzer et al. 2008). Increased microglia cell activation in the hippocampus and a concomitant increase in central TNF-α levels have been observed upon a peripheral immune challenge (Riazi et al. 2008). Similar results were obtained following peripheral administration of IL-2 (Schneider et al. 2012). Alongside elevated peripheral concentrations of inflammatory markers, both preclinical and clinical studies in depression elude to microglial activation. Investigations involving animal models of depression delineate altered expression of microglial activation markers, as well as chronicity-dependent fluctuations in microglial concentration in areas of the brain associated with mood regulation (Hinwood et al. 2012; Wohleb et al. 2012; Couch et al. 2013; Kreisel et al. 2014). Additionally, a post-mortem study also observed significant microgliosis in depressed suicide victims (Steiner et al. 2008). Not all studies agreed, as a PET scan revealed no significant differences in microglia activation in depressed individuals (Hannestad et al. 2013). Interestingly, the recent discovery of the central nervous system lymphatic system sheds new light into the possible mechanisms that may be involved in the pathogenesis of depression via neuroinflammation (Louveau et al. 2015). Regardless of the mechanisms responsible for the resulting increase in CNS inflammation following peripheral inflammation, elevated inflammatory markers within the CNS are a common finding in the pathophysiology of depression (Levine et al. 1999; Raison et al. 2006, 2009; Howren et al. 2009; Miller et al. 2009; Dowlati et al. 2010). Such unabated inflammation results in several molecular cascades associated with alterations in neurotransmitter metabolism and neuroendocrine function typically present in depression (Bakunina et al. 2015).

## Dysregulation of the endocannabinoid system in depression

### Preclinical findings

Receptors of the eCB system are widely distributed in regions of the brain markedly involved in mood regulation and mediate physiological systems associated with emotional reward, cognition and response to stress, dysregulations in all of which are frequently reported in individuals with major depression (Gorzalka and Hill 2011; Hill et al. 2012a).

The eCB system exerts its neuromodulatory effects through mediating short- and long-term forms of synaptic plasticity. Primarily involved in retrograde signalling, eCBs are synthesised and released post-synaptically in a neurotransmitter-dependent, depolarisation-induced manner. Cannabinoid receptors (CBR)s, principally located on axon terminals of glutamatergic and GABAergic neurons, are targeted by the endogenous ligands, instigating a cascade of chemical events that inhibit influx of Ca^2+^ via voltage-gated calcium channels, thereby discontinuing presynaptic release of neurotransmitters. Essential in the control of spatiotemporal eCB signalling is the intracellular enzymatic degradation of the eCBs in the presynaptic terminals, thereby terminating eCB-induced suppression of depolarisation. Key enzymes responsible for this finalising step are FAAH and MAGL (Castillo et al. 2012).

A hypoactivity of the eCB system has been observed in animal models of depression, where genetic manipulation and pharmacological interventions have provided the greatest insight into how behaviour may be affected by eCB signalling. Disruption of the eCB system resulting from CB1R knockout mice presented significant increases in depressive-like behaviour (Valverde and Torrens 2012). Similar results have been replicated with pharmacological antagonism of CB1Rs, resulting in a depressive-like phenotype (Beyer et al. 2010). Reduction in depressive-like behaviour have also been reported following FAAH inhibition (Vinod et al. 2012). Pioneering work in this field, conducted by the Piomelli group, includes the potent antidepressant-like effects elicited by a FAAH inhibitor, seemingly due to elevated levels of AEA, in the mouse models of depression, tail suspension and forced swim tests (Gobbi et al. 2005). This effect was again replicated in the chronic mild stress test in rats, while investigating the effect of chronic treatment with a FAAH inhibitor, observing a similar concomitant increase in AEA (Bortolato et al. 2007). Furthermore, overexpression of CB2Rs in mice resulted in resistance to depression following exposure to stressful situations compared to a control group (García-Gutiérrez et al. 2010). Antidepressant and anxiolytic effects ensued subsequent to intraperitoneal administration of a CB2R agonist, and this was reversed with administration of a pharmacological CB2R antagonist (Bahi et al. 2014).

Attenuation of eCB signalling is reported in chronic unpredictable stress (CUS), a well-established animal model that evokes a symptomatic profile similar to a human depressive phenotype (Willner 2005). Consequential effects of CUS in mice have delineated a decrease in CB1R function with resulting impairment of eCB-mediated retrograde signalling in the nucleus accumbens (Wang et al. 2010). Reduced eCB signalling is further supported by a significant decrease in levels of AEA in regions associated with behaviour, such as the hippocampus, hypothalamus, amygdala and ventral striatum (Hill et al. 2008a). Interestingly, despite previous reports of diminished CB1R functioning, CUS produces a contradictory increase in CB1R protein and mRNA expression in the prefrontal cortex (PFC) (Hill et al. 2008a; McLauglin et al. 2013). This increase however is suggested to be a compensatory effect of reduced eCB signalling as demonstrated by studies that reported decreased PFC AEA content (Hill et al. 2008a) and exacerbated depression following CB1R antagonist administration (McLauglin et al. 2013). Interestingly, CB1R upregulation has been documented in post-mortem studies of depressed individuals and depressed suicide victims compared to controls (Hungund et al. 2004; Choi et al. 2012).

Of note is the widely investigated involvement of eCB activity in the hypothalamic-pituitary-adrenal (HPA) axis. Dysregulation of the HPA axis is a commonly observed attribute of depression and has been implicated in its pathophysiology (Pariante and Lightman 2008; Zunszain et al. 2011). Through tonic regulation of the HPA-axis, eCB activity, mediated via CB1Rs, is responsible for maintaining basal tone (Hill and Tasker 2012b). Pharmacological blockade of the CB1Rs disrupts this tonic regulation and results in increased corticosterone levels (Patel et al. 2004; Wade et al. 2006). Likewise, CB1R KO mice exhibited depressive-like symptoms and a concomitantly higher basal adrenocorticotropic-releasing hormone level, in comparison to wild-type mice (Barna et al. 2004).

Further indication of eCB dysregulation in the aetiology of depression stems from its influence on hippocampal neurogenesis. CB2R functionality has been implicated in all key stages of neural progenitor cell cycle proliferation, differentiation and migration (Palazuelos et al. 2012; Galve-Roperh et al. 2013). Pharmacological blockade of CB1R suppressed dentate gyrus progenitor proliferation and reduced brain derived neurotrophic factor (BDNF) content in the hippocampus (Lee et al. 2009; Beyer et al. 2010). The reverse is also true, with increased stimulation of neurogenesis due to elevated BDNF following increased eCB signalling (Jiang et al. 2005; Duman and Monteggia 2006; Wyrofsky et al. 2015). It is important to consider that whilst the neurogenic theory of depression is supported by promising results in preclinical investigations, heterogeneity of clinical findings has provoked scepticism. At present, measures of neurogenesis in the human population are indirect and improvements in methodology are necessary to verify this hypothesis (Miller and Hen 2015). A reduction in hippocampal neuronal density resulted from consistent use of marijuana, the occurring process suggested to be mediated by the increased levels of THC (Landfield et al. 1988). Persistent use of marijuana also led to decreased CB1R expression (Hirvonen et al. 2012). Of relevance to depression is the aforementioned neural atrophy associated reductions in BDNF in the hippocampus, as this feature has been observed in major depression and associated animal models (Sheline et al. 1996; Santarelli et al. 2003; Lee and Kim 2010).

### Clinical findings

Clinical investigations of eCB-associated effects in the area of depression are relatively sparse. Nonetheless, findings have, for the most part, verified the eCB-depression association reported in preclinical data. For example, rimonabant, a CB1R antagonist developed for use as an anti-obesity drug, was withdrawn from the market due to serious adverse effects on mood that resulted in the onset of depression and suicidal ideation (Topol et al. 2010). Several other pharmaceuticals, namely taranabant, otenabant, ibipinabant and surinabant, also CBR antagonists, were removed earlier on, during clinical trials, for similar reasons (Wyrofsky et al. 2015). Moreover, abnormally reduced levels of ethanolamine, a precursor and metabolite of AEA, are present in the CSF of a significant proportion of individuals diagnosed with MDD, compared to healthy controls (Ogawa et al. 2015). Comparable reductions in circulating levels of 2-AG have been found in depressed patients compared to controls, which progressively decreased over the duration of the disorder (Hill et al. 2008b). Furthermore, associations have been found between gene polymorphisms in CNR1, CNR2 (genes coding for CB1R and CB2R, respectively) and FAAH genes and behaviours characteristic of depression (Zajkowska et al. 2014). However, due to the promiscuous involvement of eCB activity in numerous physiological pathways, it is important to acknowledge the great number of confounding factors, in clinical investigations of an eCB-depression association. Variances in eCB concentrations can arise from a multitude of lifestyle modifications such as exercise, diet and changes in weight as well as intake of tobacco, coffee, alcohol and cannabis. Use of pharmaceuticals is also known to influence fluctuations in eCB levels. Most critically, the use of anti-depressants, anti-psychotics and anxiolytics can upregulate the eCB system (McPartland et al. 2014).

## Crosstalk and dysregulation in the periphery

Current research delineates a complex bidirectional interaction between the peripheral eCB and immune systems, where circulating eCBs are not only affected *by* the immune system but also mediate transient effects *on* the immune response. Predominantly, CB2Rs and, to a lesser extent, CB1Rs are expressed with high and varying degrees of prevalence across the spectrum of haematopoietic cells, with B cells having the highest expression and CD4 T cells the lowest (Malfitano et al. 2014). Mechanisms that underlie eCB-mediated effects can manifest directly through communication with immune cells or indirectly via modulation of eicosanoid signalling (Rouzer and Marnett 2011). Both pathways utilise molecular cascades to manipulate activation, proliferation, secretion and apoptosis, with eventual immunoregulatory and inflammatory outcomes. Moreover, immune cells also contribute to coordinating eCB signalling through regulation of transcription, synthesis, uptake and degradation of eCB components (Pandey et al. 2009).

### Influences of eCB signalling on the immune system

Evidence suggests an involvement of eCB signalling primarily in immunosuppression. For instance, CB2R activation has been shown to attenuate inflammation in a range of inflammatory conditions from injury, inflammatory pain, hepatic injury and intestinal inflammatory disorders (Pandey et al. 2009). Furthermore, levels of circulating protein and mRNA encoding for IL-1, IL-6 and TNF-α are reduced by the administration of synthetic cannabinoids in a preclinical model of multiple sclerosis (MS) treatment (Croxford and Miller 2003). Furthermore, in an animal model of autoimmune hepatitis, Concanavalin (Con)A-induced acute hepatitis, administration of AEA has been shown to diminish hepatic injury, and this is correlated with a significant reduction in inflammatory cytokines such as TNF-α, IL-1B, IL-6, IL-9 and IL-17. The therapeutic effects of AEA are CB1 and CB2 dependent, as blockade of the receptors independently ameliorate the immunosuppressive effects (Hegde et al. 2008). However, a recent study suggests that this effect is not limited to CBR activity. Cannabidiol (CBD) is another component in marijuana; whilst it displays low affinity for CBRs, it possesses an affinity with TRPV1. CBD reduces inflammatory cytokines TNF-α, IL-2, IL-6, IL-12 and IL-17 in ConA treated mice, but this is inhibited in TPRV1 knockout (KO) mice (Hegde et al. 2011). Interestingly, the inflammatory effects exhibited in this disorder, and its preclinical model, are specifically mediated by the polyclonal activation of T cells. Hence, findings from these studies may support evidence for the expanding literature detailing eCB influences on inflammation through mediating suppression of T cell proliferation. In accordance, the eCB system is heavily implicated in regulating activation and subsequent proliferation of the lymphocytes, B cells and T cells. Evidence suggests a possible role of tonic eCB signalling that may provide an inhibitory control over spontaneous immune activation of lymphoid tissues (Pandey et al. 2009). CBRs and eCB ligands are collectively involved in the suppression of adenylate cyclase (AC) activity and by extension, cyclic adenosine monophosphate (cAMP) regulation. Lymphocyte activation requires stimulation of the cAMP signalling pathway; consequently, eCB intervention may counteract this process (Pandey et al. 2009). For example, AEA suppresses human T lymphocyte proliferation and this is dependent on CB2R action, as shown by replication of the aforementioned effect by administration of CB2R agonist and amelioration following use of a CB2R antagonist (Cencioni et al. 2010). A similar result has been demonstrated with 2-AG in mouse splenocytes, where the ligand produces a marked dose-dependent inhibition of anti-CD3 mAb-induced T cell proliferation and lipopolysaccharide (LPS)-induced B cell proliferation (Lee et al. 1995).

Therefore, application of this knowledge to reports of eCB hypoactivity in depressed individuals elicits expectation of findings depicting associated increases in lymphocyte concentrations. Surprisingly, the opposite is largely described in the literature, with reports of reduced T cell concentration in peripheral bloods of depressed patients compared to controls (Maes et al. 1995b; Zorrilla et al. 2001; Irwin and Miller 2007). However, research has yet to clearly map species-specific interactions of eCB signalling on divisions within the T cell taxonomy. Elucidation of pathways associated with non-classical receptor activation within lymphocytes is also required. Future avenues of research must work to address these issues in order understand eCB-mediated effects on lymphocyte activity in the context of depression.

### Eicosanoid signalling in eCB-mediated effects on the immune system

**Fig. 2:**
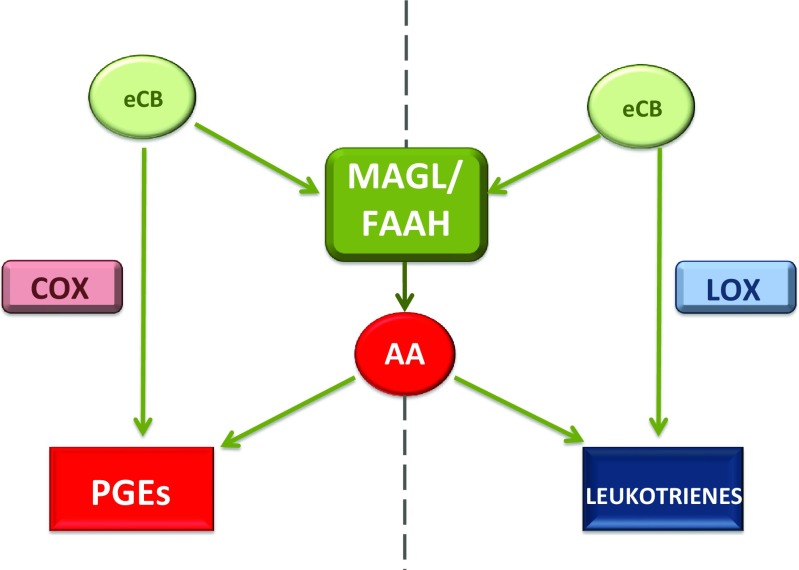
Eicosanoids, PGEs and leukotrienes, can be metabolised from two differential eCB-related pathways. 2-AG and AEA can be metabolised directly into either PGEs or leukotrienes by COX and LOX, respectively. Alternatively, degradation of eCBs can occur via enzymes, either MAGL or FAAH, into AA, which is subsequently oxidised by COX and LOX to produce PGEs and leukotrienes, respectively. Therefore, fluctuations in eCB concentration can influence eicosanoid levels

In part, eicosanoid signalling facilitates the intercommunication between eCB signalling and immunoregulation. Eicosanoids are bioactive molecules that exert a complex control over many homeostatic functions within the body; of relevance is their influence on inflammation. Biosynthesis of eicosanoids initially requires oxidation of the polyunsaturated fatty acid, arachidonic acid (AA), by the enzymes cyclooxygenase (COX) and lipoxygenase (LOX). Prostaglandins such as prostaglandin E_2_ (PGE_2_) or leukotrienes are metabolised by COX and LOX, respectively (Alhouayek et al. 2014). Both 2-AG and AEA are derivatives of AA, making them subject to the same oxidative metabolic pathways key to eicosanoid biosynthesis (Fig. 2). By extension, fluctuations in eCB signalling can impact eicosanoid signalling. As a result, eicosanoids provide a collateral method of eCB-mediated control of immune functioning (Rouzer and Marnett 2011). For example, an analogue of CBD, HU-308, a CB2R agonist that binds with high specificity, exhibits a significant anti-inflammatory effect in mice with AA-induced ear edema. Subsequent CB2R antagonist administration reverses this immunosuppression (Hanus et al. 1999; Burstein 2015). In another study, LPS-induced IL-6 production was decreased by THC, indomethacin morpholinylamide (IMMA), AEA and 2-AG in a dose-dependent manner. Interestingly, despite previously described anti-inflammatory properties of eCBs, both AEA and 2-AG did not inhibit COX-2 production of PGE_2_; moreover, 2-AG, contrary to THC, IMMA and AEA, caused a subsequent increase in iNOS-dependent NO (itself an inflammatory agent) production, thus providing an immunoenhancing effect. Discrepancies within the results seen in 2-AG, PGE_2_ and NO production were understood to be the result of increased metabolic degradation of 2-AG to AA, which in turn served as a substrate for catalysing increased COX-2 production of PGE_2_, known to potentiate NO production (Chang et al. 2001). This study demonstrates that inflammation is a constant balance between pro-inflammatory and anti-inflammatory substances and eCB and eicosanoid signalling play homeostatic roles in its regulation.

An alternative pathway of enzymatic oxidation of AA by LOX provides immunosuppressing properties that mitigate those observed by COX-produced metabolites. *N*-arachindonoylglycine, another endogenous eicosanoid, is an AEA metabolite (Bradshaw et al. 2009). This cannabinoid analogue has anti-inflammatory effects, probably thanks to its ability to promote the release of AA, successively increasing LOX-catalysis of AA to lipoxin A4 (LXA_4_) (Burstein et al. 2011). Increased concentrations of LXA_4_ are associated with the resolution phase of an acute inflammatory response, in which it induces inflammatory cell apoptosis (Bannenberg and Serhan 2010). Given the elevated inflammatory profile observed in depression and the immunoregulating properties of lipoxins and their relationship with eCBs, it would be interesting to explore a potential association between depression and lipoxins. No study to date has directly investigated this avenue of research.

Interestingly, PGE_2_ has been implicated in depression. Elevated levels of PGE_2_ in plasma, serum and saliva have been measured in patients with MDD (Lieb et al. 1983; Ohishi et al. 1988; Song et al. 1998). PGE_2_ has been demonstrated in inflammatory conditions to upregulate phosphodiesterase-4 (Oger et al. 2015), which in turn inhibits downstream formation of BDNF (Logan 2003), which itself is implicated in depression.

### Influences of immunocompetent cells on eCB signalling

Biochemical evidence also suggests the immune system plays a profound role in the regulation of eCB signalling. Immune stimuli, such as LPS, have demonstrated that immune cell activation can also modulate eCB signalling by increasing expression of CBRs (Klein et al. 2003). Of relevance, lymphoid CBR and AEA are also elevated in patients suffering from MS, where pro-inflammatory cytokines are a pathogenic component of the disease (Sospedra and Martin 2005); following 1 year of treatment with the anti-inflammatory IFN-β, levels of AEA and expression of CBR progressively decrease (López et al. 2014).

Elucidation of underlying mechanisms in immunocompetent cells demonstrates an endogenous control and modulation over key stages in eCB functioning. A preclinical study of murine macrophages demonstrates their capacity to produce both AEA and 2-AG, intriguingly through differential pathways. AEA synthesis was observed to be dependent on LPS-induced transcription of proteins either directly responsible for the synthesis of AEA or involved in the activation of proteins responsible for the synthesis of AEA. NF-кB appeared to be a key signalling molecule in the LPS-induced transcriptional pathways. In contrast, LPS-induced increases in platelet-activating factor (PAF) are the primary intermediate of 2-AG synthesis in macrophages, dependent on PAF receptor activation (Liu et al. 2003).

Immune cells are also involved in termination of eCB signalling through direct uptake and degradation of AEA and 2-AG. AEA in vitro has been shown to accumulate in peripheral immune cells in a concentration-dependent manner (Hillard and Jarrahian 2003). Following LPS administration, increased peripheral lymphatic AEA levels are seen, probably as the result of reduced FAAH expression, thus demonstrating the presence of degradative enzymes within human peripheral lymphocytes (Maccarrone et al. 2001).

## Crosstalk between the eCB and immune systems in the CNS

**Fig. 3:**
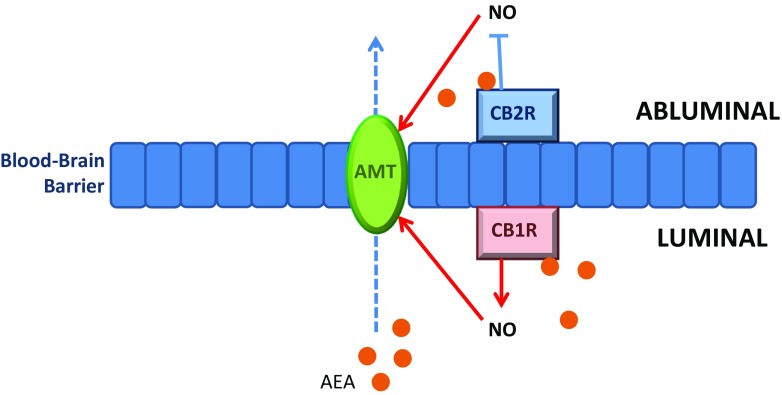
Differential expression of CB1R and CB2R on the luminal and abluminal membranes, respectively, may lead to unidirectional transport of AEA. CB1R increases nitric oxide (*NO*) production, which in turn induces activity of a selective AEA membrane transporter (*AMT*). Increased levels of AEA are then transported across the BBB. CB2R negates NO production and therefore suppresses AMT activity. Thus, transport of abluminal AEA across the BBB is reduced

Peripheral eCB signalling can be reflected in the brain through transportation of eCBs across the blood-brain barrier (BBB) via endothelial cell membranes of brain microvessels. Studies report a differential expression of CB1R and CB2R on the luminal and abluminal membranes, respectively, as well as opposing function: CB1R increases NO production and thus augments AEA membrane transporter (AMT) activity, and CB2R supresses AMT activity by negating NO production (Maccarrone et al. 2005). Taken together, these findings indicate a possible unidirectional transportation of AEA across the BBB (Centonze et al. 2008) (Fig. 3). A similar neuroprotective role has been observed following administration of two highly selective CB2R agonists, which reduce TNF-α-induced upregulation of several genes within the primary human brain microvascular endothelial cells that constitute the BBB. Using functional assays, CB2R stimulation results in BBB protection proceeding inflammatory insult and diminished monocyte migration across the BBB (Persidsky et al. 2015).

Whilst the presence of CB1Rs in the CNS has been well characterised, the role and expression of CB2Rs has been the object of controversy. However, recent understanding of methodological flaws in detecting CB2R expression in the CNS has improved current methods of detection. As such supporting evidence has been on the rise. Since identifying the presence of CBRs on immunocompetent cells within the CNS, implications of intercommunication between the eCB and immune systems have become increasingly prominent. Microglia, the resident immune cells of the CNS, is of particular relevance due to their considerable neuroimmune regulatory properties. CB1Rs are located on microglia, neuronal terminals and astrocytes, with CB2Rs largely expressed in microglial as well as glial cells (Stella 2010). Moreover, microglial cells are not only the primary source and target for inflammatory mediators (Czirr and Wyss-coray 2012) but cultured astrocytes and microglia are also capable of 2-AG and AEA production (Stella 2009). Chronically activated microglial cells have been increasingly associated with neurodegenerative disorders and neuroinflammatory progression (Streit et al. 2004). Likewise, upregulation of CB2Rs has been found in activated microglia and in neuroinflammatory disorders, such as MS, motor neurone disease, Down syndrome and Alzheimer’s disease (Rom and Persidsky 2013). Such a coexisting dysregulation observed in both systems suggests an underlying communication and implicates potential impairment of its interaction in neurological disorders where inflammation is central to its pathogenesis, a feature now growing in recognition as relevant to depression.

### Influences of eCB signalling on the immune system

**Fig. 4:**
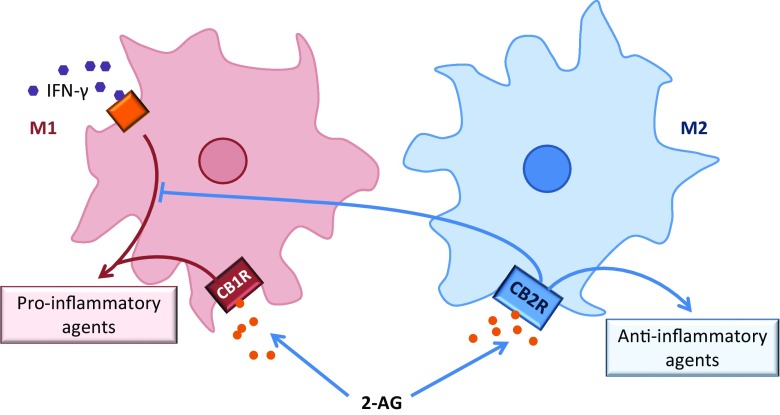
Differential binding of 2-AG to CBRs on microglia elicits differential immunomodulatory effects, dependent on the state of microglial activation. Binding of CB2R on M2 state microglia is associated with increased concentrations of anti-inflammatory agents. CB2R-2AG binding inhibits IFN-γ-induced microglial cell expression of proinflammatory agents. 2-AG binding to CB1R in M1-activated state microglia, results in increased pro-inflammatory mediators

Through use of CB2R agonists and transgenic mice, CB2Rs have been described as proficient neuroprotective mediators and are involved in reduced BBB endothelial cell stimulation and inflammatory responses (Fernández-ruiz et al. 2006). Their role in neuroinflammation has been characterised as largely suppressive, consistent with notions of neuroprotective properties, through actions that directly alter the balance of pro-inflammatory and anti-inflammatory mediators (Rom and Persidsky 2013). In accordance, recent studies have established a predominantly anti-inflammatory effect of microglial CB2R stimulation (Fernández-ruiz et al. 2006). In vitro and in vivo studies report similar findings of cannabinoid-induced increases in glial cell secretion of the anti-inflammatory cytokines IL-4 and IL-10 (Molina-Holgado et al. 1998). Successive investigation also found that CB2R signalling inhibits IFN-γ-induced mouse microglial cell expression of the pro-inflammatory mediators, iNOS and CCR2, a gene encoding for a receptor associated with monocyte infiltration (Racz et al. 2008). Two states of microglial cell activation are documented, a classic activated state (M1) and the alternative state (M2) (Cherry et al. 2014). M1 polarisation is associated with cytotoxic pro-inflammatory secretion of TNF-α, IL-1β and IL-6, whereas M2 is considered to have anti-inflammatory and neurotrophic properties, releasing IL-10 and BDNF (Ma et al. 2015). Recent indications of a CB2R role in mediating a shift from M1 state to M2 have been described. Following pretreatment with the CB2R agonist AM1241, inflammation is attenuated, with a concomitant enhanced release of anti-inflammatory and neurotrophic factors. Moreover, expression of iNOS, a marker for M1 activity, decreases, whilst expression of M2 marker, Arg-1, increases. AM1241 effects are ameliorated after administration of CB2R antagonist and protein kinase C (PKC) inhibitor, indicating firstly that the effects are CB2R mediated but also that PKC activity may facilitate the effects (Ma et al. 2015). Such results support the immunosuppressive observations seen of CB2R activation in the brain. Similarly, exogenous 2-AG administration in acute experimental autoimmune encephalomyelitis, a preclinical model, produces increased microglia population as well as M2 shifting. A recent mechanism proposed by Nakagawa and Chiba (2014) alludes to a differential immunomodulatory action of 2-AG dependent on the state of microglial activation and CBR. 2-AG binding to CB1R in M1-activated state microglia results in increased proinflammatory mediators, whereas 2-AG binding to CB2R on M2-polarised microglia elevates the anti-inflammatory cytokine IL-10 and the proresolving factor lipoxin A4 (Nakagawa and Chiba 2014) (Fig. 4). Further examination into the influences of the eCB system on microglial polarisation need to be carried out in order to provide a more comprehensive support.

Impaired functionality of 2-AG-CB2R binding may contribute, in part, to the onset of depression. A study of note, conducted by Onaivi et al. (2008), found that a particular polymorphism (Q63R) of the CB2R gene is of significantly higher incidence in Japanese depressed patients than controls. Interestingly, this polymorphism has been shown to compromise the activation of the receptor in mice, with reports that 2-AG binding with CB2-Q63R produce significantly lesser maximum responses than in wild-type mice (Carrasquer et al. 2010). Additionally, chronic administration of minocycline, which has anti-inflammatory action, attenuates depressive-like behaviours in the olfactory bulbectomised animal model of depression. It also shows a concomitant downregulation of CD11, a marker of M1 activation, increased expression of the M2 microglia state marker, MRC2, and a shift from pro-inflammatory to anti-inflammatory cytokines (Burke et al. 2014). Taken together, the aforementioned evidence provides support for the hypothesis of impaired M2 polarisation in individuals suffering with depression, possibly due to reduced 2-AG-CB2R binding. Nonetheless, more research is needed to determine the effects of various eCB-CBR binding on microglial secretion of inflammatory markers, in the context of depression. Further to this, despite substantial evidence to support the dynamic plasticity in microglial function, only two studies, to date, have investigated an associated microglia polarisation skew in depression. More critically, reported findings were contradictory (Hannestad et al. 2013; Burke et al. 2014). However, a recent compelling study reported significant anti-inflammatory effects of commonly prescribed antidepressants, fluoxetine and escitalopram, mediated by a shift from M1 microglia polarisation to M2 alternative state activation (Su et al. 2015). This suggests the efficacy of these therapeutic interventions may, in part, be attributable to altered microglial plasticity. Further investigation is greatly warranted in order to ascertain the implications posed, by evidence of microglia polarisation, to our understanding of the pathophysiology underlying depression.

Additional implications of 2-AG in the suppression of neuroinflammation are supported by the observation that 2-AG, as well as AEA, suppresses LPS-induced TNF-α secretion by microglial cells (Facchinetti et al. 2003). Microglial cells secrete 2-AG in response to elevated neuroinflammation caused by injury (Panikashvili et al. 2001; Stella 2004). Furthermore, 2-AG increases microglial cell proliferation in a CB2R-dependent manner (Carrier et al. 2004).

Interestingly, the eicosanoid system seems to be implicated in CNS endocannabinoid immune-related communication. Increases in 2-AG and attenuation of free AA concentrations have been reported in mice treated with the MAGL inhibitor, JZL184 (Long et al. 2009). Genetic and pharmacological inhibition of MAGL in LPS administered mice resulted in reduced prostaglandins PGE_2_, PGD_2_, PGF_2a_ as well as pro-inflammatory cytokines IL-1α, IL-1β, IL-6 and TNF-α in the brain, despite LPS administration. Consistent with the notion of eicosanoid involvement, these immunosuppressive effects are replicated by COX-1 blockade but are not reversed by a CB1R antagonist (Nomura et al. 2011).

### Influences of the immune system on eCB signalling

The communication in the CNS, as in the periphery, is bidirectional, and extensive research has demonstrated not only effects of the eCB system on immunomodulation but also immunoregulatory effects of the immune system on eCB signalling. For example, upon LPS administration, CB1R expression is significantly reduced, probably due to reduced CB1R protein (Hsieh et al. 1999). In particular, CB1R immunoreactivity is diminished in the hippocampus and dorsal bed nucleus of the terminal stria terminalis in mice, areas associated with major depression and stress/reward pathways. However, CB1R mRNA is increased after LPS administration, indicating inflammation-induced adaptive regulation at the protein level as opposed to transcriptional dysregulation (Hu et al. 2012). The same study reported that LPS decreases expression of CB1R in CA3 pyramidal layer glutamatergic nerve terminals of the hippocampus. These findings may indicate a reduction in CB1R dependent suppression of glutamatergic synaptic transmission. Such overactivation of glutamatergic pathways can result in increased excitoxic damage and subsequent hippocampal atrophy, changes that have associations with mood disorders (Jorge et al. 2007; Zunszain et al. 2011).

## Concluding remarks

**Fig. 5:**
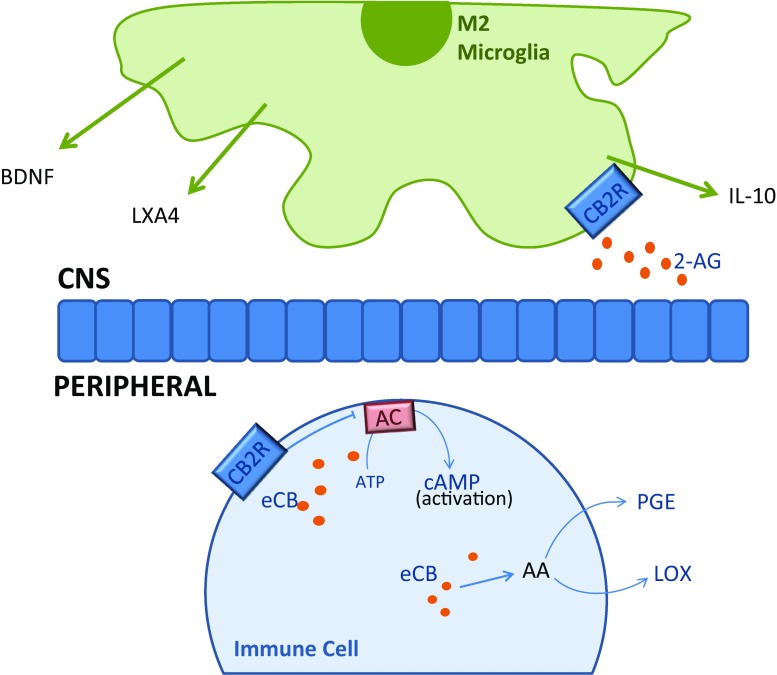
Peripheral: eCB action on CB2Rs results in suppression of immune cell activation by inhibiting AC conversion of adenosine tri-phosphate (*ATP*) to cAMP. eCBs can be metabolised in to arachidonic acid (*AA*) which can subsequently be converted to inflammatory molecule, prostaglandins (PGE), or anti-inflammatory agent, leukotrienes (*LOX*) CNS: 2-AG induces M2-state microglia to secrete anti-inflammatory agents; BDNF, LXA4 and IL-10, mediated by CB2Rs

This nascent field of research implicates an intricate intercommunication between the peripheral and central eCB and immune systems in the pathogenesis of depression. Findings depict a multifaceted communication whereby immunocompetent and eCB-related cells can both influence the suppression and enhancement of the other’s activity. Typical functioning of the peripheral eCB system involves a tonic signalling control over spontaneous immune cell activation, mediated by cAMP pathways, thereby suppressing an inflammatory response. Similarly, eCB interactions with neural constituents provide key neuroprotective effects, suggested to be, in part, due to increased anti-inflammatory microglial M2-state cells through 2-AG-CB2R functioning. Moreover, an interaction with the eicosanoid signalling system influences the balance of pro- and anti-inflammatory agents: prostaglandin and leukotrienes, respectively. By extension, dysfunction of the eCB system has potential to chronically activate the inflammatory response and exacerbate neurodegeneration, both characteristics frequently observed in depression (Fig. 5).

Comprehensive understanding of the biochemistry that manifests with depression warrants clarification on the impact of cell type, concentration and cellular environment and receptor density on eCB-immune processing. Elucidation of these factors will increase the potential for effectual therapeutic intervention towards a more personalised approach.

**Table:** 

Summary
• Inflammatory component in depression - Elevated levels of inflammatory signalling proteins in plasma and serum taken from individuals with depression - Increased inflammatory markers within the CNS - Altered microglial activity in preclinical and clinical studies of depression - Associated changes in inflammation with typically reported abnormalities in physiological processes implicated in depression; neurotransmitter metabolism, neuroendocrine function and neuroprogression
• Role of the endocannabinoid (eCB) system in depression - Reported hypoactivity of the eCB system in animal models of depression - Manipulation of the eCB system results in associated changes in depressive-like behaviour in preclinical studies - HPA axis dysregulation, a commonly documented attribute of depression, can be produced by alterations of the eCB system - eCB system is implicated in stages of neurogenesis
• Peripheral crosstalk and dysregulation of the eCB-immune system and its associations with depression - eCB activity is involved in immunosuppression through tonic regulation of cAMP production - 2-AG and AEA (eCBs) can be metabolised to produce eicosanoids. Dependent on the degradative enzyme, inflammatory or anti-inflammatory molecules, PGE or LOX, respectively, are produced - Abnormal concentrations of PGE_2_ have been associated with depression - Activated immune cells modulate eCB levels through their production, uptake and degradation
- Central crosstalk and dysregulation of the eCB-immune system and its associations with depression - eCBs are largely neuroprotective, due to their suppression of neuroinflammation - Suppression of neuroinflammation is suggested to be the result of eCB influences on microglial state polarisation. - Possible association between reduced 2-AG-CB2R binding on M2 (alternative state microglia) and depression - Immune insult to the CNS alters levels of CB1R expression in areas associated with major depression - Reduced CB1R expression can lead to hippocampal atrophy, a feature reported in mood disorders.
• Future directions Peripheral focus - Studies that address depression and eCB and immune system crosstalk, conjointly, both in the periphery and CNS - Clarification of eCB and lymphocyte interaction, in the context of depression - Outline specific interactions of eCB signalling with different species included in the T cell taxonomy. - Elucidate potential disparities in pathways associated with *non-classical* receptor activation of lymphocytes. - Further study of eicosanoid, in particular lipoxin, concentrations in individuals with depression CNS focus - Further examination into influences of the eCB system on microglial polarisation - Investigations into an association between microglial polarisation state and depression - Effects of various eCB-CBR binding on microglia secretion of inflammatory markers and whether this effect is associated with depression.
